# Enhanced EGFP Fluorescence Emission in Presence of PEG Aqueous Solutions and PIB_1000_-PEG_6000_-PIB_1000_ Copolymer Vesicles

**DOI:** 10.1155/2013/329087

**Published:** 2013-07-10

**Authors:** Noor Muhammad, Nadezda Kryuchkova, Tamara Dworeck, Francisco Rodríguez-Ropero, Marco Fioroni

**Affiliations:** ^1^Department of Biotechnology (Biology VI), RWTH Aachen University, Worringerweg 1, 52074 Aachen, Germany; ^2^Department of Biotechnology and Genetic Engineering, Kohat University of Science and Technology, Kohat 26000, Pakistan; ^3^Department of Ecology and Evolution, University of Lausanne, 1015 Lausanne, Switzerland; ^4^Department of Biology, RWTH Aachen University, Worringerweg 1, 52074 Aachen, Germany; ^5^Technische Universität Darmstadt, Center of Smart Interfaces, Petersenstraße 32, 64287 Darmstadt, Germany; ^6^Konrad-Müller Straße 17, 52249 Eschweiler, Germany

## Abstract

An EGFP construct interacting with the PIB_1000_-PEG_6000_-PIB_1000_ vesicles surface reported a ~2-fold fluorescence emission enhancement. Because of the constructs nature with the amphiphilic peptide inserted into the PIB core, EGFP is expected to experience a “pure” PEG environment. To unravel this phenomenon PEG/water solutions at different molecular weights and concentrations were used. Already at ~1 : 10 protein/PEG molar ratio the increase in fluorescence emission is observed reaching a plateau correlating with the PEG molecular weight. Parallel experiments in presence of glycerol aqueous solutions did show a slight fluorescence enhancement however starting at much higher concentrations. Molecular dynamics simulations of EGFP in neat water, glycerol, and PEG aqueous solutions were performed showing that PEG molecules tend to “wrap” the protein creating a microenvironment where the local PEG concentration is higher compared to its bulk concentration. Because the fluorescent emission can be perturbed by the refractive index surrounding the protein, the clustering of PEG molecules induces an enhanced fluorescence emission already at extremely low concentrations. These findings can be important when related to the use of EGFP as reported in molecular biology experiments.

## 1. Introduction 

The wild type green fluorescent protein (GFP) was first isolated from the jellyfish *Aequorea victoria*. It is a 27 kDa protein, composed of 238 amino acids that are arranged in 11 antiparallel *β*-sheets forming *β*-barrel geometry. In the center of the *β*-barrel structure is located the covalently bound, p-hydroxybenzylidene-imidazolidinone based fluorophore [1] formed by a posttranslational autocatalytic cyclization of Ser-65, Tyr-66, and Gly-67 residues [2, 3]. This makes the GFP a flexible biological indicator and marker with broad applications; that is, GFP can be fused to a great number of proteins and allows their intracellular localization and detection [4]. Furthermore a variety of GFP derivatives have been either discovered in other organisms like corrals (i.e., DsRed from Discosoma) [5] or derived by mutation [6], nowadays listing more than 200 GFP-like proteins with emission wavelengths covering practically the entire visual spectrum. One of the GFP-like proteins derived by mutation is the so-called EGFP (“enhanced GFP”), a GFP mutant with improved characteristics, especially suitable for the expression in mammalian cells. EGFP contains two amino acid substitutions (Phe64 to Leu and Ser65 to Thr) that lead to better thermal stability and a 35-times higher emission amplitude [7, 8]. Due to the aforementioned applications, the dependence of the EGFP or GFP brightness on external variables is an important topic [9].

In a previous study regarding an amphiphilic peptide anchor for the decoration of polymersome surfaces, the EGFP was fused to the peptide anchor to function as a reporter molecule [10]. The study revealed that the detected fluorescence of the peptide-EGFP fusion in presence of PIB_1000_-PEG_6000_-PIB_1000_ (PIB = polyisobutylene, PEG = polyethylene glycol) polymersomes was stronger than that of free peptide-EGFP fusion. In order to further analyze this interesting finding, the effects of the PIB_1000_-PEG_6000_-PIB_1000_ polymer, PEG with different molecular weight and glycerol on the fluorescence of free EGFP has been carried out.

Furthermore as the chromophore emission is a function of the folding state [11] by means of molecular dynamics simulations we have tried to understand the microscopic effect of the different environments (water, water/glycerol, and water/PEG solutions) on the EGFP dynamics.

## 2. Materials and Methods

### 2.1. Experimental Section

All chemicals were of analytical grade or higher and purchased from Sigma-Aldrich Chemie (Taufkirchen, Germany) and AppliChem (Darmstadt, Germany) if not stated otherwise. Protein concentrations were determined using the standard BCA kit (Pierce Chemical Co, Rockford, IL, USA).

### 2.2. Overexpression and Purification of EGFP-C-His

The plasmid pEGFP for the expression of EGFP-C-His was purchased from BD Biosciences Clontech (Heidelberg, Germany). The size of pEGFP is 3.4 kb; it carries an ampicilline resistance, lacI promoter, and pUC origin of replication.

pEGFP carries a red-shifted variant of wild-type GFP (GFP mut1) with a C-terminal His-tag, which has been optimized for brighter fluorescence. GFP mut1 contains the double amino acids substitution of Phe-64 to Leu and Ser-65 to Thr [8]. Cells of *E. coli* XL2 Blue have been transformed with plasmid pEGFP. For EGFP-C-His expression cells were grown on liquid LB medium containing ampicillin at 37°C and 250 rpm. The culture was induced by the addition of 1 mM IPTG at an OD600 of 0.6. Cells were harvested after an additional 16 h of incubation under the same conditions. Cells were disrupted by using a pressure homogenizer (1500 bar, 2 cycles; Avestin Emulsiflex, Mannheim, Germany) and the protein extraction reagent B-Per (Pierce Chemical Co, Rockford, IL, USA). EGFP-C-His was isolated and purified by applying the cleared cell lysate to a Protino Ni-IDA column (2000 Protino His-Tag Protein purification Kit, Macherey-Nagel, Düren, Germany), and purification was carried out according to the kit manual. To avoid protease digestion of the protein during purification 1 mM PMSF was added to the lysate.

### 2.3. SDS-PAGE

 The purified EGFP was analyzed by SDS-PAGE [12], and protein was visualized by Coomassie Brilliant blue R-250 staining.

### 2.4. Measurement of EGFP Fluorescence in Solutions of Different PEG MW and Glycerol with Varying Concentrations

 EGFP solutions in glycerol, PEG_600_, PEG_6000_, PEG_8000_, and PEG_20000_ were prepared. PEG and glycerol concentrations were 10^−3^, 10^−4^, 10^−5^, 10^−6^, 10^−7^, and 10^−8^ mol/L. The EGFP concentration was fixed at 10^−8^ mol/L in all samples. Fluorescence intensity of samples including EGFP in plain water was measured using Tecan Infinite M 1000 (Tecan Group Limited, Mannedorf, Switzerland) and Greiner black flat bottom microtiter plates (Frickenhausen, Germany) (excitation wavelength: 488 nm, emission wavelength: 509 nm).

### 2.5. Measurements of EGFP Fluorescence in Presence of PIB_1000_-PEG_6000_-PIB_1000_ Copolymer Vesicles

The PIB_1000_-PEG_6000_-PIB_1000_ based tri-block copolymer was obtained from BASF AG (Germany). Experimental procedures for vesicles production and characterization have been reported in a previous work [10].

### 2.6. Theoretical Section. EGFP, PEG, and Glycerol Models

 The EGFP crystal structure (PDB entry: 2Y0G) was obtained from the Protein Data Bank (http://www.pdb.org/).  Figures were designed with the Discovery Studio Visualizer 2.0 program version, obtainable at http://accelrys.com/products/discovery-studio/visualization/discovery-studio-visualizer.html. The PEG model was adopted from Fischer et al. [13], while the glycerol model was taken from the GROMACS library. The EGFP chromophore was selected in its neutral form [14].

### 2.7. Simulations Details

 All the MD simulations were performed using GROMACS 4.0.7 [15] molecular dynamics simulation package (http://www.gromacs.org/). Three systems were simulated, that is, EGFP in water, EGFP in a 0.006 M glycerol water solution, and EGFP in a 0.004 M polyethylene glycol (PEG) solution. Each simulated system was placed in the center of a dodecahedron box. Explicit water molecules represented by the SPC model [16] were used. Details of each simulated system are summarized in Table 1. 

EGFP consists of 226 residues (2329 atoms). Periodic boundary conditions were applied, and a time step of 2 fs was used for the numerical integration of the equations of motion. Atomic coordinates were saved every 5 ps. Simulations were conducted at a constant temperature of 300 K and a constant pressure of 1 bar. Solvent, protein, sodium ions, and PEG or glycerol were independently coupled to a temperature bath, with a coupling constant of *τ*_*T*_ = 0.02 ps, by a V-rescale thermostat [17]. An isotropic pressure coupling for the water solution simulations was used, with a coupling constant of *τ*_*P*_ = 1.0 ps and a compressibility of 4.5∗10^−5^ bar^−1^ by a Berendsen barostat [18]. The GROMACS Force Field (ffG53a6) [19], in which aliphatic carbons are treated using the united atom representation, was used. Energy minimizations were performed using a steepest descent algorithm followed by a constrained molecular dynamics. Constraints on the protein backbone atoms were applied by a harmonic potential with a force constant of 10 kJ mol^−1^ Å^−2^ and slowly diminished to 0 kJ mol^−1^ Å^−2^ within the equilibration time. Bond distances were constrained using the LINCS algorithm [20] while the van der Waals interactions were modeled using a 6–12 Lennard-Jones potential with a cutoff at 1 nm. The electrostatic interactions were calculated by using the Particle Mesh Ewald algorithm (PME) [21] with a cutoff of 1 nm for the direct space calculation. The reciprocal space calculation was performed using a fast Fourier transform algorithm. A simulation time of 50 ns was selected to satisfy the rotational correlation time of the end-to-end vector of the polyethylene glycol [13] with an MW = 600. All the simulations were analyzed using the tools for analysis implemented in GROMACS. 

## 3. Results and Discussion

**Figure 1:**
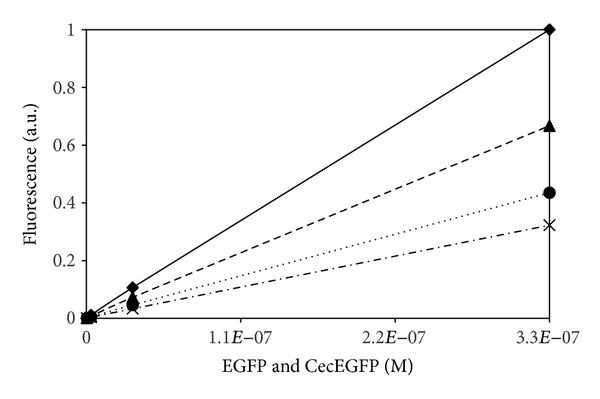
Fluorescence intensity of EGFP and the Cecropin-10Ala-EGFP construct [10] in water solution (EGFP: dashed; construct: dashed-dot) and in PIB_1000_-PEG_6000_-PIB_1000_ solution (0.33 mM) (EGFP: line; construct: dot).

By a “serendipitous” finding, while setting a calibration curve to understand how many EGFP molecules were present on a polymersome surface, a clear correlation between the presence of vesicles and an increased fluorescence was revealed (Figure 1).

In fact though the construct shows always a lower fluorescence intensity compared to the single EGFP both in water or in the water-polymer solution—probably due to a partial EGFP unfolding when linked to the Cecropin peptide—the data obtained for neat water clearly show, for both EGFP and the construct, a lower intensity when compared to the corresponding water/polymer solution. 

In case of the construct, while the amphiphilic peptide has been shown to be inserted into the polymersome membrane, the linked EGFP was well expected to “float” on the hydrophilic surface made of PEG [10]. Interestingly also in the case of the free EGFP the polymer increases the fluorescence. However because the number of EGFP molecules embedded into a vesicle/micelles is but a tiny fraction of the total EGFP present in water solution and because the protein was shown not to interact with the polymer vesicles [10] (the SEC data show practically no shift between the pure protein or when solved in water/polymer solution), the increased fluorescence has been explained by the interaction of the protein with few low molecular weight polymer chains, granted by the polydispersity of the sample (PDI = 1,85).

As a further understanding of the phenomenon, a series of experiments where PEG was the only polymer present in aqueous solution were performed to be compared with glycerol/aqueous solutions to check whether and how a similar low molecular weight molecule affects EGFP [22].

**Figure 2:**
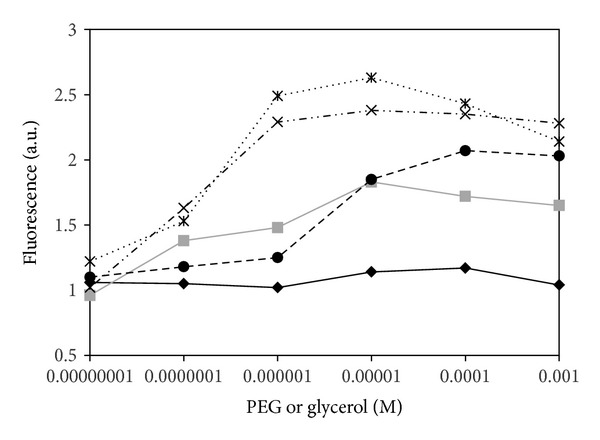
Fluorescence intensity of EGFP at a fixed molar concentration (10^−8^ [M]) in presence of varying concentrations of glycerol (continuous black line, diamonds) and PEG at different MW: MW = 600: continuous grey line, squares; MW = 6000: dot-dashed, crosses; MW = 8000: ultrafine dotted, stars; MW = 20000: dots, filled circles.

In Figure 2 data are shown where the concentration of EGFP was maintained constant [10^−8^] while PEG and glycerol were changed in such a way that for each molecule of EGFP there are 1 : 10 : 10^2^ : 10^3^ : 10^4^ : 10^5^ molecules of PEG or glycerol.

The experimental findings (Figure 2) clearly show an EGFP fluorescence enhancement when polyethylene glycol is present, while in glycerol solutions the emission increases only slightly at higher concentrations. 

Most importantly PEG induces a higher fluorescence intensity already at very low concentrations ~10^−7^ [M] corresponding to a 1 : 10 molecular ratio between the protein and the polymer. Moreover the effect reaches a plateau depending on the PEG molecular weight.

By definition, the quantum yield *f*_(*F*)_ of a fluorophore is defined by *f*_(*F*)_ = *k*_*F*_/(*k*_*F*_ + *k*_ic_ + *k*_is_ + *k*_*q*_), where  *k*_*F*_ = fluorescence constant,  *k*_ic_ = internal conversion,  *k*_is_ = intersystem crossing, and *k*_*q*_ = quenching mechanism/molecule. Considering the previous relation the EGFP fluorescent enhancement is due to a higher chromophore quantum yield, where the internal conversion, intersystem crossing, and quenching are less efficient to deploy energy. In completely different systems, a six- to ten-fold enhancement of the fluorescence emission from GFP was reported near a silver surface, showing higher photostability and reduced blinking [23]. However some previous studies performed in a similar system on the fluorescence decay of EGFP in presence of glycerol/water solutions were conducted by Suhling et al. [22]. The fluorescence decay of EGFP was shown dependent from the refractive index of the medium, where a higher refractive index decreases the fluorescence lifetime inducing a higher fluorescence. On the other hand decreasing the refractive index of the medium a higher fluorescence lifetime and consequentially a lower brightness were reached. However, in the experiments described in the present article, the glycerol or block copolymer concentrations are much lower compared to the work of Suhling. 

Shifting the attention to the microscopic region surrounding the protein, Molecular Dynamics studies were performed to correlate the protein dynamics to the enhanced fluorescence. previous molecular dynamics studies on GFP [24] or EGFP [25] have been conducted. The protein itself is a very robust *β*-barrel protein able to inner-bury the chromophore assuring through a complex net of hydrogen bonds, the planarity of the fluorescent p-hydroxybenzylidene-imidazolidinone by the *τ* and *ϕ* dihedrals (See Supporting Information in Supplementary Material available online at http://dx.doi.org/10.1155/2013/329087). Such a condition is necessary if fluorescence must be preserved as well described by a series of GFP chromophore synthetic models dissolved in water, glycerol, and dioxane, where the torsional barrier mainly affects fluorescence efficiency [26]. As first hypothesis, the PEG environment was thought to induce a higher planarity to the chromophore “caged” in a more rigid protein structure. However the enhanced emission already starts when ~10 PEG molecules are in presence of 1 EGFP molecule. Furthermore checking the DSSP [27], RMSD, and RMSF of the protein backbone and the chromophore rigidity, the hypothesis seems not to be valid (see Supporting Information).

**Figure 3:**
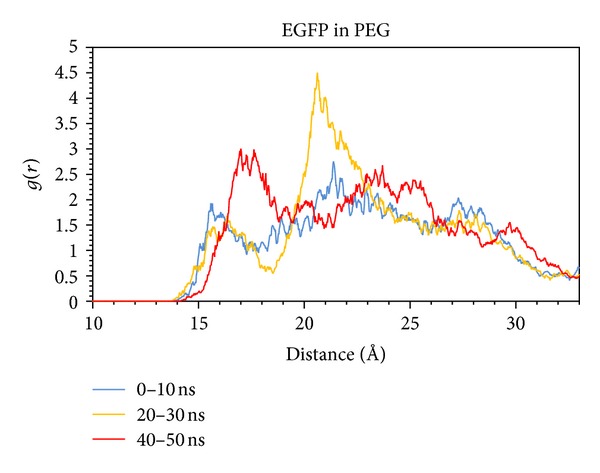
Molecular centers of mass radial distribution function of PEG.

**Figure 4:**
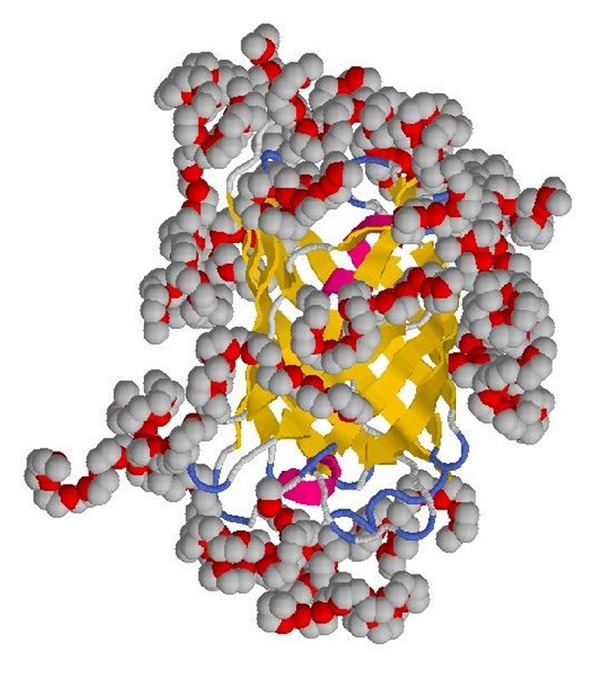
Final snapshot obtained after 50 ns of molecular-dynamics simulations showing the wrapped PEG molecules embedding EGFP.

Though the secondary and tertiary structure of the protein itself seem not to be affected much within the simulated different environments, the behavior of the PEG polymer shows a peculiar “clustering” around the protein (Figures 3 and 4), due to the presence of well-defined peaks as found in radial distribution function, enhancing the local concentration of PEG.

This phenomenon has been already found in a class of fluorinated alcohols/water solutions, where the fluorinated cosolvents tend to be clustered around the protein (with a bulk solution of 30% v/v the protein “sees” an 80% v/v solution) [28]. This explains also the fluorescence saturation effect though after a molecular weight of 10000 g/mol PEG induces a protein destabilization [29] explaining the rapid decay of the fluorescence intensity.

Based on the previous finding the fluorescence enhancement at very low PEG concentrations can be linked to the PEG-enriched microenvironment surrounding the protein. 

As previously reported one consequence of such a phenomenon relates to the refractive index of the medium embedding the protein itself: a higher refractive index induces a lower decay time [22, 30, 31] with the consequence of a higher brightness or, from the microscopic point of view, a higher quantum yield. 

On the other hand, glycerol shows a slight effect on the fluorescence intensity starting only at higher concentrations in comparison to PEG (Figure 2). Furthermore the radial distribution function (see Supporting Information) does not show any special clustering or organized shell surrounding the protein, suggesting the EGFP fluorescence intensity is affected only when the “bulk” concentration changes the solution refractive index.

Though simulations based on EGFP in presence of the PIB_1000_-PEG_6000_-PIB_1000_ block copolymer have not been conducted, the same mechanism can be proposed in the light of the experimental data previously shown. In fact the enhanced fluorescence emission of the EGFP construct in presence of the PIB_1000_-PEG_6000_-PIB_1000_ vesicles (Figure 1) shows how the EGFP environment is PEG based floating on the polymersome hydrophilic surface while the amphiphilic peptide is inserted into the PIB hydrophobic region as an anchor (as experimentally reported) [10].

In conclusion all findings confirm that EGFP can be a sensitive probe to detect environments with different refractive index [22, 29].

## 4. Conclusions

The EGFP fluorescence emission intensity has been found to be a function of the PEG (polyethylene glycol), glycerol, and the triblock copolymer PIB_1000_-PEG_6000_-PIB_1000_ (PIB = polyisobutylene) concentration. The fluorescence intensity increases already at very low protein/PEG molar ratio (1 : 10) with glycerol showing the same trend though at higher molar ratio (>1 : 100). Performing molecular dynamics simulations of EGFP in neat water, glycerol and PEG aqueous mixtures, a clustering of PEG molecules wrapping the EGFP has been found. Though this phenomenon is known for fluorinated solvents, the effect seems to relate to the EGFP-enhanced brightness. Particularly as observed for PEG, the local PEG higher density increases the local refractive index surrounding the protein perturbing the fluorescence lifetime and, as a consequence, increasing the protein brightness (higher refractive index, lower fluorescence lifetime, and higher emission).

In a different system where the EGFP was linked to an anchor peptide to interact with polymersomes constituted by the PIB_1000_-PEG_6000_-PIB_1000_ block copolymer the same trend has been noticed. Due to the nature of the system, with the amphiphilic peptide inserted into the (hydrophobic) PIB copolymer core and the EGFP floating on the (hydrophilic) PEG surface, the protein emission enhances due to the local high concentration of PEG chains. 

On the other hand, glycerol does not show any special clustering or organized shell surrounding the protein, suggesting the EGFP fluorescence intensity is affected only when the “bulk” concentration changes the solution refractive index.

The obtained results clearly show how the sensitivity of the EGFP to the local environment can be used to obtain valuable information on the local physicochemical conditions with interesting possible applications.

## Supplementary Material

Chromophore MD parameterization and topology, protein DSSP, RMSD and RMSF analysis, RMSD of the *ϕ* and *τ* chromophore angles and g(r) of Glycerol/EGFP center of masses.

## Figures and Tables

**Table 1:** Details of the simulated systems. Total number of particles (*N*_atoms_), total number of water molecules (*N*_water_), total number of glycerol molecules (*N*_glyc_), total number of polyethylene glycol molecules (*N*_PEG_), and number of sodium atoms (*N*_Na^+^_).

System	*N*_atoms_	*N*_water_	*N*_glyc_	*N*_PEG_	*N*_Na^+^_
Water	22788	6818	—	—	5
Glycerol	20586	3902	394	—	5
PEG	18381	5063	—	22	5
