# Supplementary material for: Enhanced EGFP Fluorescence Emission in Presence of PEG Aqueous Solutions and PIB1000-PEG6000-PIB1000 Copolymer Vesicles
Source: Biomed Res Int. 2013 Jul 10;2013:329087. doi: 10.1155/2013/329087 (PMC3723060; doi:10.1155/2013/329087)
Supplement: Supplementary file 1 — Chromophore MD parameterization and topology, protein DSSP, RMSD and RMSF analysis, RMSD of the ϕ and τ chromophore angles and g(r) of Glycerol/EGFP center of masses. [file 329087.f1.doc]

**Supporting Information**

Enhanced EGFP fluorescence emission in presence of PEG aqueous solutions and PIB1000-PEG6000-PIB1000 copolymer vesicles.

Noor Muhammad, Nadezda Kryuchkova, Tamara Dworeck, Francisco Rodríguez-Ropero and Marco Fioroni

Corresponding Author email: [rodriguez@csi.tu-darmstadt.de](mailto:rodriguez@csi.tu-darmstadt.de) and [mfioroni@lycos.com](mailto:mfioroni@lycos.com)

**Content**

**Chromophore MD parameterization and topology S3-S6**

**DSSP plots S7**

**Protein backbone RMSD and RMSF (single residue) S8**

**RMSD of the β-sheet and α-helix domains S9**

**RMSD and φ and τ chromophore angles S10**

**Radial Distribution Function Glycerol/EGFP S11**

**Chromophore MD parameterization and Topology**

Two initial configurations were taken into account, *i.e.* *cis* and *trans*. Structures full optimization were carried out using the Gaussian 09 program, [15](#_ENREF_15) using the B3LYP[16](#_ENREF_16)^-^[17](#_ENREF_17) functional combined with the aug-cc-pVDZ basis set. [18](#_ENREF_18) The *cis* conformer is the lowest in energy. Furthermore *cis* and *trans* conformations are separated by a barrier of 7.67 kcal/mol.

Chromophore bonding and Lennard-Jones interactions were parameterized according to the GROMOS96 G53a6 [19](#_ENREF_19) while charges were obtained by an *ab initio* estimation using the CHelpG [20](#_ENREF_20) procedure under the Gaussian 09 program on the previously optimized *cis* structure. Topology of the chromophore file is included in the supporting information.


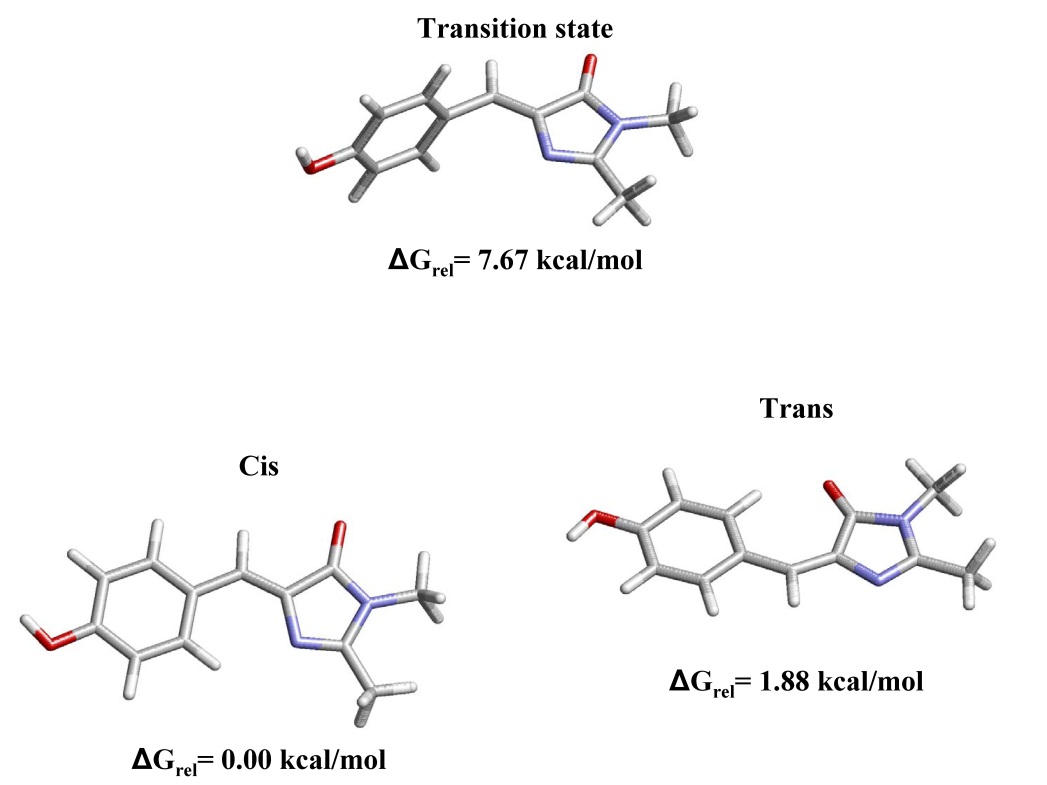
The topology file was tested in a 0.1 ns long Molecular Dynamics (MD) in vacuum at 600 K on short peptide formed by Leu-Chromophore-Val-Gln.

**Figure S1.** Optimized structures at the B3LYP/ aug-cc-pVDZ of the chromophore in the *cis* and *trans* conformations. The transition state and their ΔG_rel_ have been also included.

**References:**

15. M. J. Frisch, G. W. T., H. B. Schlegel, G. E. Scuseria, M. A. Robb, J. R. Cheeseman, G. Scalmani, V. Barone, B. Mennucci, G. A. Petersson, H. Nakatsuji, M. Caricato, X. Li, H. P. Hratchian, A. F. Izmaylov, J. Bloino, G. Zheng, J. L. Sonnenberg, M. Hada, M. Ehara, K. Toyota, R. Fukuda, J. Hasegawa, M. Ishida, T. Nakajima, Y. Honda, O. Kitao, H. Nakai, T. Vreven, J. A. Montgomery, Jr., J. E. Peralta, F. Ogliaro, M. Bearpark, J. J. Heyd, E. Brothers, K. N. Kudin, V. N. Staroverov, R. Kobayashi, J. Normand, K. Raghavachari, A. Rendell, J. C. Burant, S. S. Iyengar, J. Tomasi, M. Cossi, N. Rega, J. M. Millam, M. Klene, J. E. Knox, J. B. Cross, V. Bakken, C. Adamo, J. Jaramillo, R. Gomperts, R. E. Stratmann, O. Yazyev, A. J. Austin, R. Cammi, C. Pomelli, J. W. Ochterski, R. L. Martin, K. Morokuma, V. G. Zakrzewski, G. A. Voth, P. Salvador, J. J. Dannenberg, S. Dapprich, A. D. Daniels, Ö. Farkas, J. B. Foresman, J. V. Ortiz, J. Cioslowski, and D. J. Fox *Gaussian 09*, Revision A.1; Gaussian, Inc.: Wallingford, CT, 2009.

16. Becke, A. D., A new mixing of hartree-fock and local density-functional theories. *Journal of Chemical Physics* **1993,** *98* (2), 1372-1377.

17. Lee, C. T.; Yang, W. T.; Parr, R. G., Development of the colle-salvetti correlation-energy formula into a functional of the electron-density. *Physical Review B* **1988,** *37* (2), 785-789.

18. Dunning, T. H., *J. Chem. Phys.* **1989,** *90*, 1007.

19. Oostenbrink, C.; Villa, A.; Mark, A. E.; Van Gusteren, W. F., *J. Comput. Chem.* **2004,** *25*, 1656.

20. Breneman, C. M.; Wiberg, K. B., *J. Comput. Chem.* 1990, *11*, 361.

**Table S1:** Chromophore Gromacs topology file

| [ MFC ] |  | |  |  |  |
| --- | --- | --- | --- | --- | --- |
| [ atoms ] |  | | Charges | Group |  |
| N | N | | -0.28000 | 0 |  |
| H | H | | 0.28000 | 0 |  |
| CA1 | CH1 | | 0.35000 | 1 |  |
| CB1 | C | | 0.45000 | 1 |  |
| CG1 | CH3 | | 0.00000 | 1 |  |
| OG1 | O | | -0.48000 | 1 |  |
| C1 | CR1 | | 0.18000 | 1 |  |
| N2 | NR | | -0.52000 | 1 |  |
| N3 | N | | -0.08000 | 1 |  |
| C2 | C | | 0.38000 | 2 |  |
| O2 | O | | -0.38000 | 2 |  |
| CA2 | CR1 | | 0.10000 | 1 |  |
| CA3 | CH2 | | 0.00000 | 3 |  |
| C | C | | 0.38000 | 4 |  |
| O | O | | -0.38000 | 4 |  |
| CB2 | C | | -0.28000 | 5 |  |
| HB2 | HC | | 0.10000 | 5 |  |
| CG2 | C | | 0.18000 | 5 |  |
| CD1 | C | | -0.10000 | 6 |  |
| HD1 | HC | | 0.10000 | 6 |  |
| CD2 | C | | -0.10000 | 7 |  |
| HD2 | HC | | 0.10000 | 7 |  |
| CE1 | C | | -0.20000 | 8 |  |
| HE1 | HC | | 0.10000 | 8 |  |
| CE2 | C | | -0.20000 | 8 |  |
| HE2 | HC | | 0.10000 | 8 |  |
| CZ | C | | 0.36000 | 8 |  |
| OH | OA | | -0.56000 | 8 |  |
| HC | H | | 0.40000 | 8 |  |
|  |  | |  |  |  |
| [ bonds ] |  | |  |  |  |
| N | H | | gb_2 |  |  |
| N | CA1 | | gb_20 |  |  |
| CA1 | C1 | | gb_26 |  |  |
| CA1 | CB1 | | gb_26 |  |  |
| CB1 | OG1 | | gb_4 |  |  |
| CB1 | CG1 | | gb_26 |  |  |
| C1 | N2 | | gb_9 |  |  |
| C1 | N3 | | gb_9 |  |  |
| N3 | CA3 | | gb_9 |  |  |
| CA3 | C | | gb_26 |  |  |
| C | O | | gb_4 |  |  |
| C | +N | | gb_9 |  |  |
| N3 | C2 | | gb_9 |  |  |
| C2 | O2 | | gb_4 |  |  |
| C2 | CA2 | | gb_9 |  |  |
| CA2 | N2 | | gb_9 |  |  |
| CA2 | CB2 | | gb_9 |  |  |
| CB2 | HB2 | | gb_3 |  |  |
| CB2 | CG2 | | gb_26 |  |  |
| CG2 | CD1 | | gb_15 |  |  |
| CG2 | CD2 | | gb_15 |  |  |
| CD2 | CE2 | | gb_15 |  |  |
| CE2 | CZ | | gb_15 |  |  |
| CZ | OH | | gb_4 |  |  |
| CZ | CE1 | | gb_15 |  |  |
| CE1 | CD1 | | gb_15 |  |  |
| OH | HC | | gb_1 |  |  |
| CD1 | HD1 | | gb_3 |  |  |
| CD2 | HD2 | | gb_3 |  |  |
| CE1 | HE1 | | gb_3 |  |  |
| CE2 | HE2 | | gb_3 |  |  |
|  |  | |  |  |  |
| [ angles ] |  | |  |  |  |
| TYR like |  | |  |  |  |
| CD2 | CE2 | | CZ | ga_26 |  |
| CE2 | CZ | | CE1 | ga_26 |  |
| CZ | CE1 | | CD1 | ga_26 |  |
| CE1 | CD1 | | CG2 | ga_26 |  |
| CD1 | CG2 | | CD2 | ga_26 |  |
| CG2 | CD2 | | CE2 | ga_26 |  |
| CE2 | CZ | | OH | ga_26 |  |
| CE1 | CZ | | OH | ga_26 |  |
| CE1 | CD1 | | HD1 | ga_24 |  |
| CG2 | CD1 | | HD1 | ga_24 |  |
| CD1 | CE1 | | HE1 | ga_24 |  |
| CZ | CE1 | | HE1 | ga_24 |  |
| CD2 | CE2 | | HE2 | ga_24 |  |
| CZ | CE2 | | HE2 | ga_24 |  |
| CE2 | CD2 | | HD2 | ga_24 |  |
| CG2 | CD2 | | HD2 | ga_24 |  |
| HIS like |  | |  |  |  |
| CD1 | CG2 | | CB2 | ga_56 |  |
| CD2 | CG2 | | CB2 | ga_14 |  |
| CB2 | CA2 | | N2 | ga_57 |  |
| CB2 | CA2 | | C2 | ga_58 |  |
| CG2 | CB2 | | CA2 | ga_55 |  |
| CG2 | CB2 | | HB2 | ga_24 |  |
| CA2 | CB2 | | HB2 | ga_24 |  |
| -C | N | | H | ga_31 |  |
| H | N | | CA1 | ga_17 |  |
| -C | N | | CA1 | ga_30 |  |
| CA3 | C | | +N | ga_18 |  |
| CA3 | C | | O | ga_29 |  |
| +N | C | | O | ga_32 |  |
| CB1 | CA1 | | C1 | ga_12 |  |
| OG1 | CB1 | | CA1 | ga_29 |  |
| OG1 | CB1 | | CG1 | ga_29 |  |
| CB1 | CA1 | | N | ga_18 |  |
| N | CA1 | | C1 | ga_30 |  |
| CA1 | CB1 | | CG1 | ga_12 |  |
| CA1 | C1 | | N2 | ga_36 |  |
| CA1 | C1 | | N3 | ga_36 |  |
| C1 | N3 | | C2 | ga_6 |  |
| C1 | N2 | | CA2 | ga_6 |  |
| N2 | C1 | | N3 | ga_6 |  |
| N3 | C2 | | CA2 | ga_6 |  |
| N3 | C2 | | O2 | ga_32 |  |
| CA2 | C2 | | O2 | ga_29 |  |
| N2 | CA2 | | C2 | ga_6 |  |
| C2 | N3 | | CA3 | ga_6 |  |
| C1 | N3 | | CA3 | ga_6 |  |
| N3 | CA3 | | C | ga_6 |  |
| CZ | OH | | HC | ga_11 |  |
| CE1 | CZ | | OH | ga_26 |  |
|  |  | |  |  |  |
| [ impropers ] | | |  |  |  |
| TYR like |  | |  |  |  |
| CZ | OH | | CE2 | CE1 | gi_1 |
| CG2 | CD1 | | CD2 | CB2 | gi_1 |
| CG2 | CD2 | | CE2 | CZ | gi_1 |
| CD2 | CE2 | | CZ | CE1 | gi_1 |
| CE2 | CZ | | CE1 | CD1 | gi_1 |
| CZ | CE1 | | CD1 | CG2 | gi_1 |
| CE1 | CD1 | | CG2 | CD2 | gi_1 |
| CD2 | CE2 | | CG2 | HD2 | gi_1 |
| CE2 | CZ | | CD2 | HE2 | gi_1 |
| CE1 | CZ | | CD1 | HE1 | gi_1 |
| CD1 | CE1 | | CG2 | HD1 | gi_1 |
| HIS like |  | |  |  |  |
| C | O | | CA3 | +N | gi_1 |
| CA1 | CB1 | | C1 | N | gi_4 |
| C1 | N2 | | N3 | CA1 | gi_1 |
| CA2 | N2 | | C2 | CB2 | gi_1 |
| C2 | CA2 | | N3 | O2 | gi_1 |
| N3 | C2 | | C1 | CA3 | gi_1 |
| N2 | CA2 | | C2 | N3 | gi_1 |
| N2 | C1 | | N3 | C2 | gi_1 |
| N3 | C2 | | C1 | CA3 | gi_1 |
| Double bond | | |  |  |  |
| CB2 | | CG2 | CA2 | HB2 | gi_1 |
| C2 | | CA2 | CG2 | CD2 | gi_1 |
|  | |  |  |  |  |
| [ dihedrals ] | |  |  |  |  |
| TYR like | |  |  |  |  |
| CA2 | | CB2 | CG2 | CD1 | gd_42 |
| CB2 | | CG2 | CD2 | CE2 | gd_43 |
| HIS like | |  |  |  |  |
| -CA | | -C | N | CA1 | gd_4 |
| -C | | N | CA1 | C1 | gd_19 |
| N3 | | CA3 | C | +N | gd_20 |
| N | | CA1 | CB1 | CG1 | gd_1 |
| N | | CA1 | C1 | N2 | gd_20 |
| CA1 | | C1 | N2 | CA2 | gd_4 |
| CA1 | | C1 | N3 | C2 | gd_4 |
| O2 | | C2 | N3 | CA3 | gd_16 |
| C1 | | N2 | CA2 | C2 | gd_20 |
| O2 | | C2 | N3 | C1 | gd_19 |
| O2 | | C2 | CA2 | N2 | gd_19 |
| C1 | | N3 | CA3 | C | gd_4 |
| CA3 | | C | +N | +CA | gd_4 |
| C | | +N | +CA | +C | gd_4 |
| CE1 | | CZ | OH | HC | gd_20 |
| N2 | | CA2 | CB2 | CG2 | gd_42 |


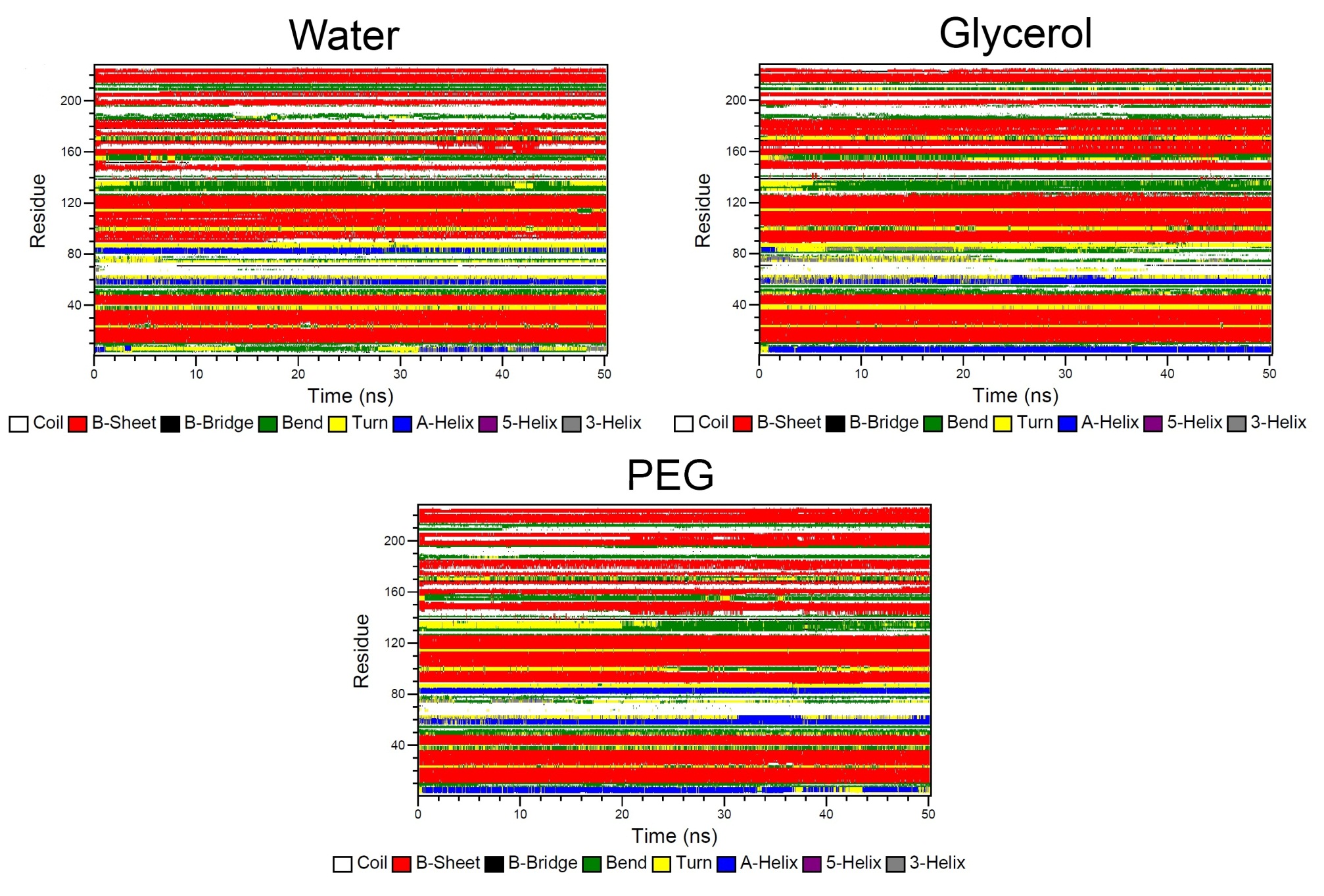


**Figure S2.** DSSP^1^ plots of EGFP in different solvent environments.

1 Determination of Secondary Structure in Proteins: W. Kabsch and C. Sander, Dictionary of protein secondary structure: Pattern recognition of hydrogen-bonded and geometrical features. *Biopolymers,* **22,** 2577-2637 (1983)


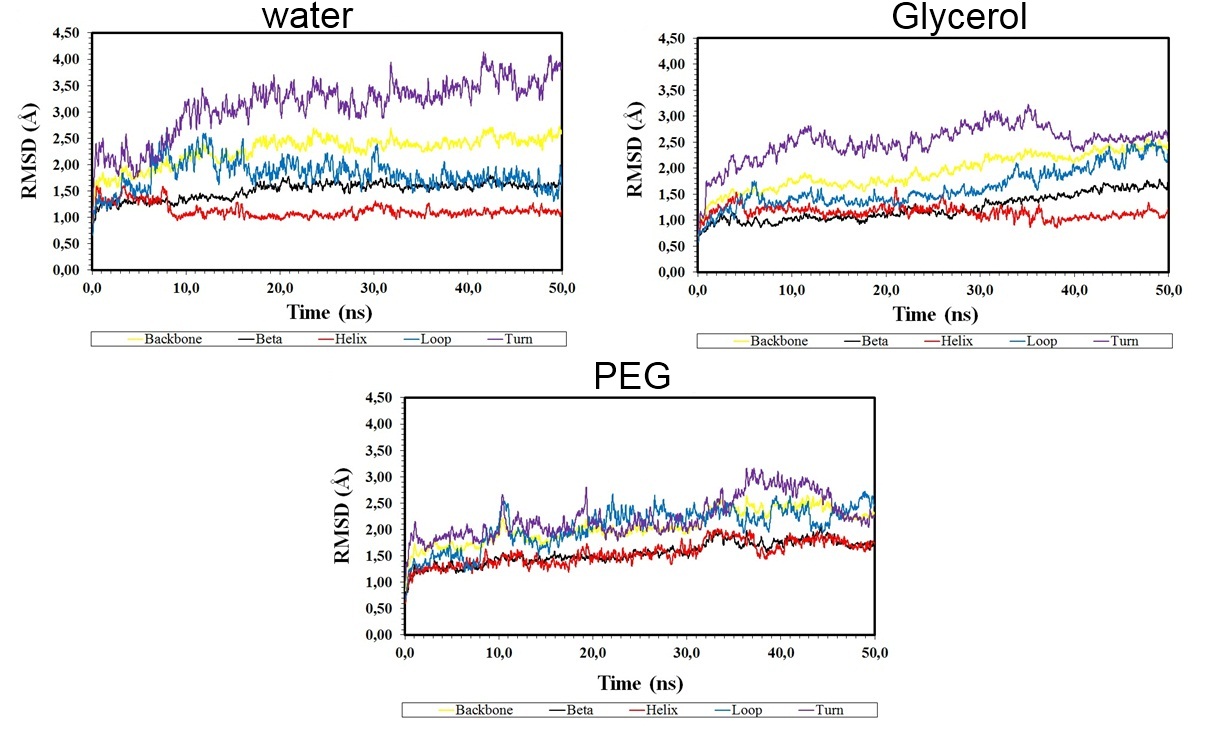


**Figure S3.** RMSD plots of the EGFP backbone in different solvent environments.


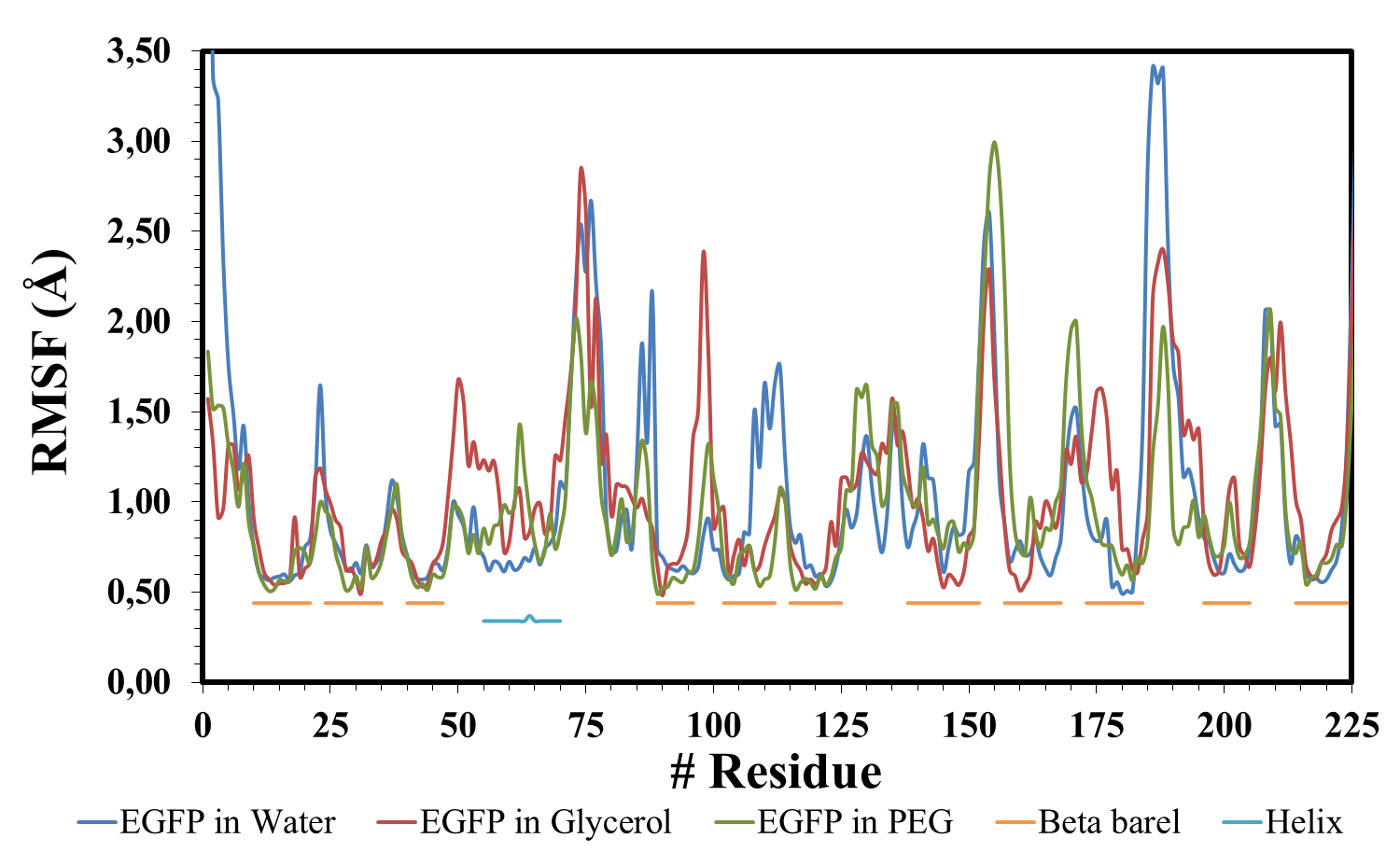


**Figure S4.** RMSF plots of the single EGFP residues in different solvent environments.


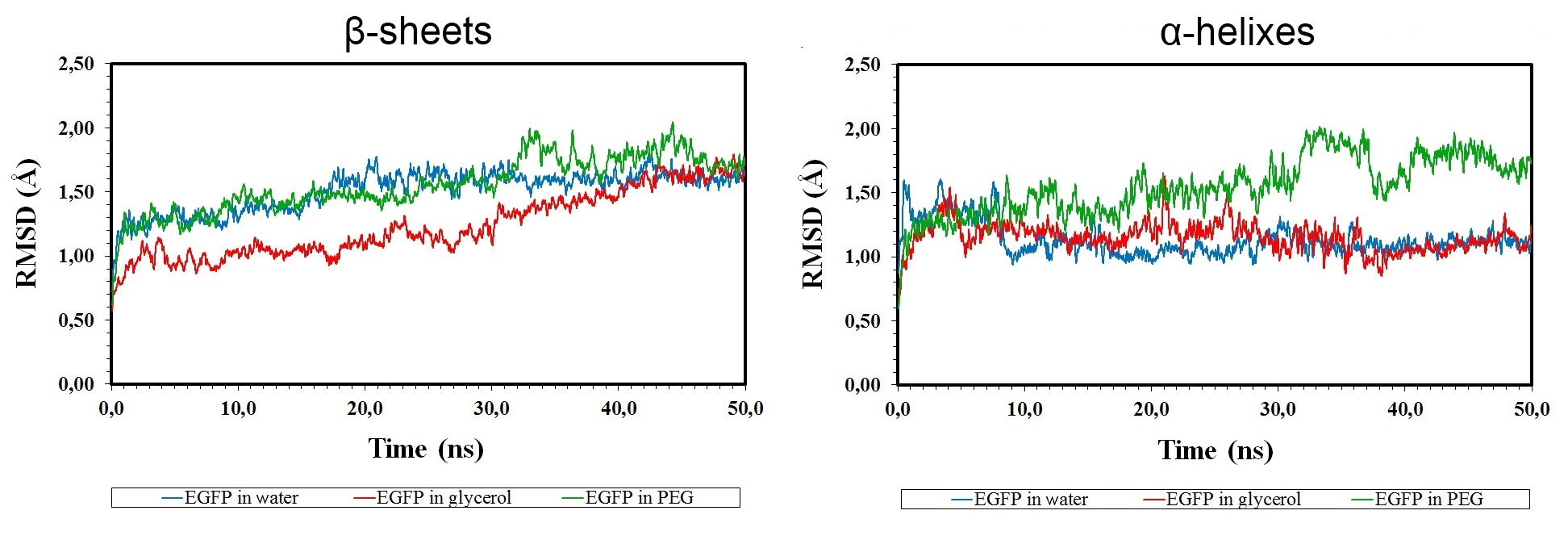


**Figure S5.** RMSD plots of the α- and β-domains within the EGFP in different solvent environments.


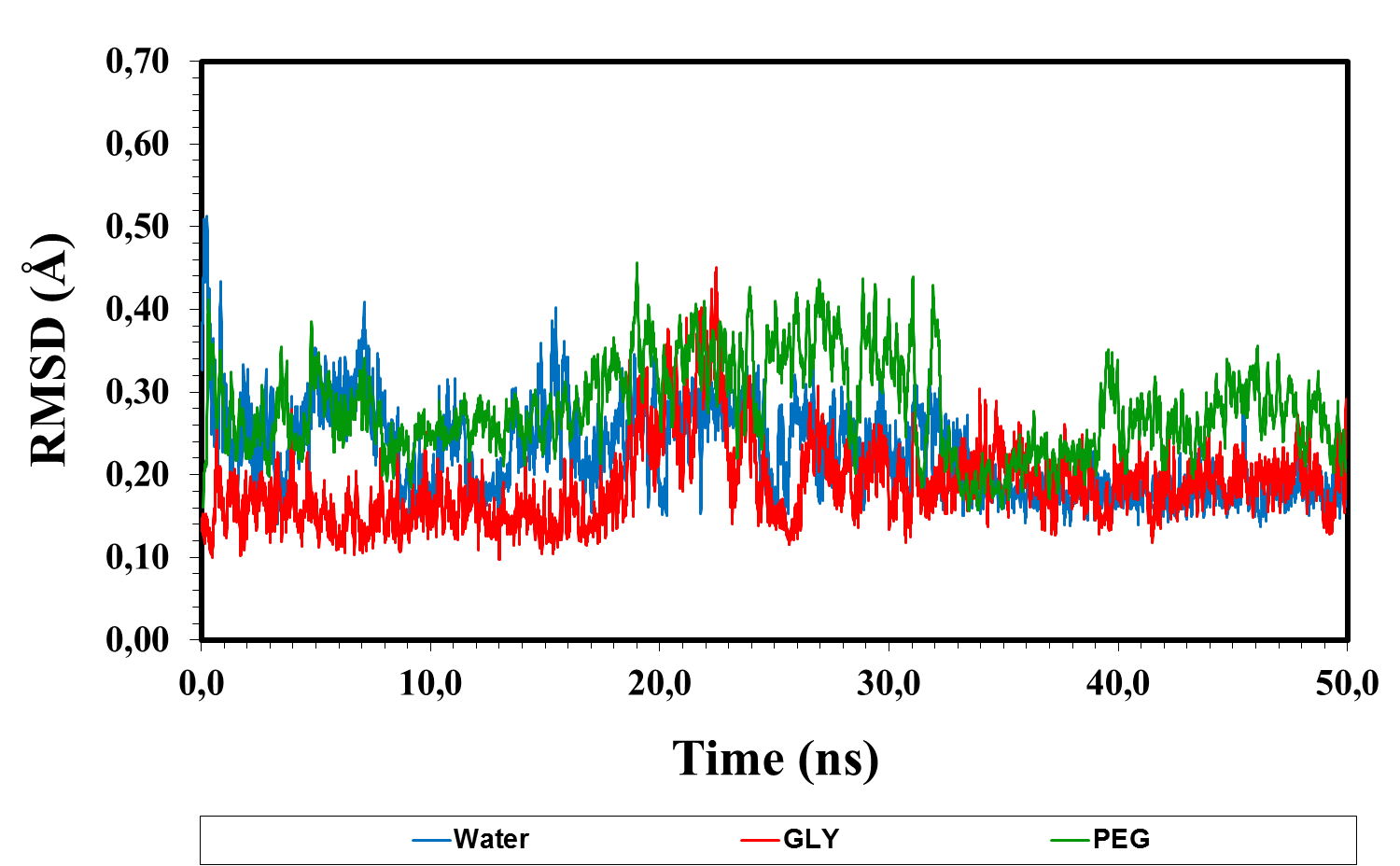

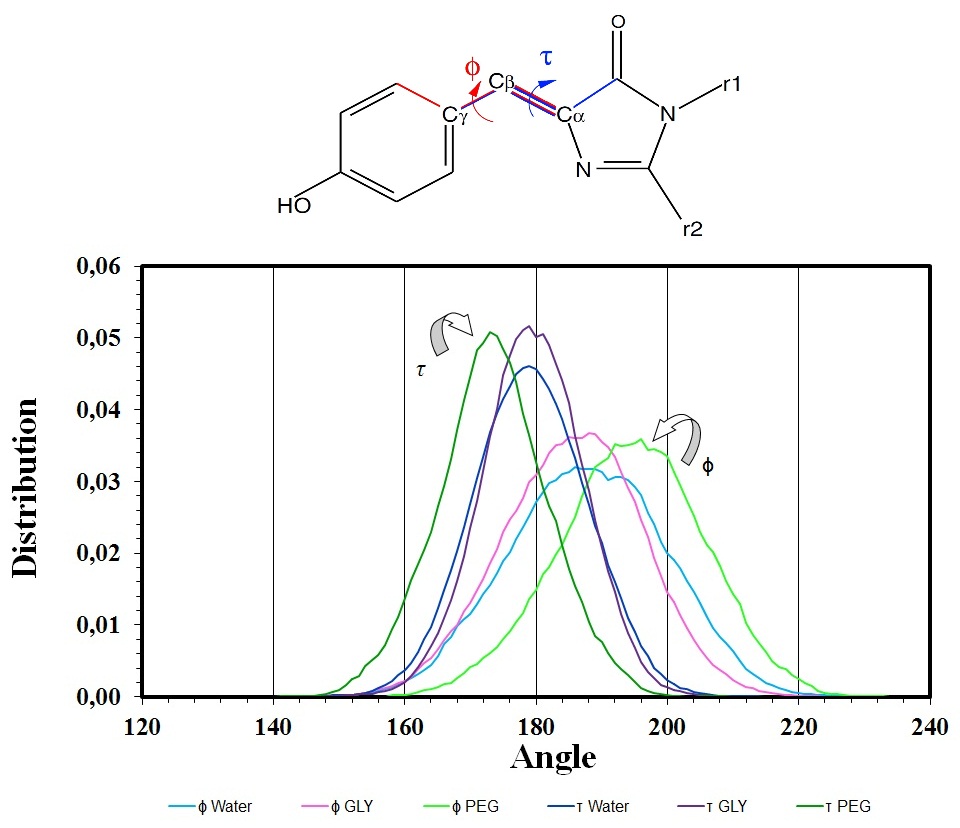

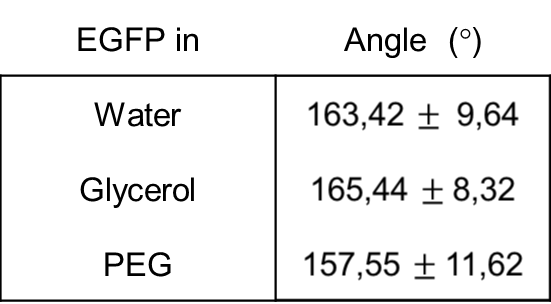


**Figure S6.** Chromophore RMSD plot and τ, φ angles distribution with the EGFP in different solvent environments.


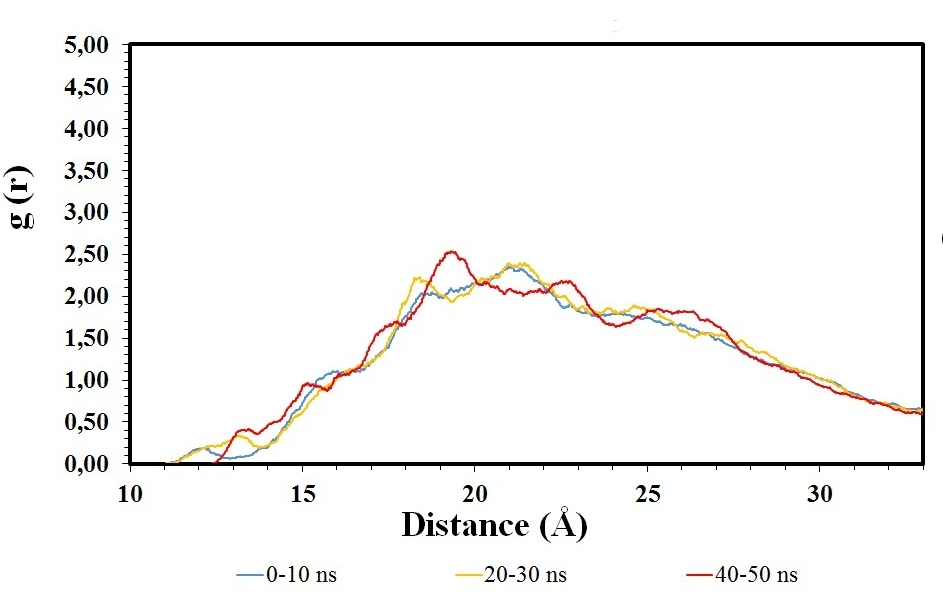


**Figure S7.** Radial distribution function of the glycerol centres of mass toward the centre of mass of EGFP.
